# Pyruvate Kinase Deficiency in Sub-Saharan Africa: Identification of a Highly Frequent Missense Mutation (G829A;Glu277Lys) and Association with Malaria

**DOI:** 10.1371/journal.pone.0047071

**Published:** 2012-10-17

**Authors:** Patrícia Machado, Licínio Manco, Cláudia Gomes, Cristina Mendes, Natércia Fernandes, Graça Salomé, Luis Sitoe, Sérgio Chibute, José Langa, Letícia Ribeiro, Juliana Miranda, Jorge Cano, João Pinto, António Amorim, Virgílio E. do Rosário, Ana Paula Arez

**Affiliations:** 1 Centro de Malária e outras Doenças Tropicais, Unidade de Parasitologia Médica, Instituto de Higiene e Medicina Tropical, Universidade Nova de Lisboa, Lisboa, Portugal; 2 Centro de Investigação em Antropologia e Saúde (CIAS), Universidade de Coimbra, Coimbra, Portugal; 3 Faculdade de Medicina da Universidade Eduardo Mondlane, Maputo, Mozambique; 4 Banco de Sangue do Hospital Central de Maputo, Maputo, Mozambique; 5 Departmento de Hematologia, Centro Hospitalar de Coimbra, Coimbra, Portugal; 6 Hospital Pediátrico David Bernardino, Luanda, Angola; 7 Centro Nacional de Medicina Tropical, Instituto de Salud Carlos III, Madrid, Spain; 8 Instituto de Patologia e Imunologia Molecular da Universidade do Porto (IPATIMUP), Porto, Portugal; 9 Faculdade de Ciências da Universidade do Porto, Porto, Portugal; Université Pierre et Marie Curie, France

## Abstract

**Background:**

Pyruvate kinase (PK) deficiency, causing hemolytic anemia, has been associated to malaria protection and its prevalence in sub-Saharan Africa is not known so far. This work shows the results of a study undertaken to determine PK deficiency occurrence in some sub-Saharan African countries, as well as finding a prevalent PK variant underlying this deficiency.

**Materials and Methods:**

Blood samples of individuals from four malaria endemic countries (Mozambique, Angola, Equatorial Guinea and Sao Tome and Principe) were analyzed in order to determine PK deficiency occurrence and detect any possible high frequent PK variant mutation. The association between this mutation and malaria was ascertained through association studies involving sample groups from individuals showing different malaria infection and outcome status.

**Results:**

The percentage of individuals showing a reduced PK activity in Maputo was 4.1% and the missense mutation G829A (Glu277Lys) in the PKLR gene (only identified in three individuals worldwide to date) was identified in a high frequency. Heterozygous carrier frequency was between 6.7% and 2.6%. A significant association was not detected between either PK reduced activity or allele 829A frequency and malaria infection and outcome, although the variant was more frequent among individuals with uncomplicated malaria.

**Conclusions:**

This was the first study on the occurrence of PK deficiency in several areas of Africa. A common PKLR mutation G829A (Glu277Lys) was identified. A global geographical co-distribution between malaria and high frequency of PK deficiency seems to occur suggesting that malaria may be a selective force raising the frequency of this 277Lys variant.

## Introduction

Infectious diseases have been one of the major causes of mortality during most of human evolution. For many diseases, mortality and hence reproductive success are influenced by certain individual genotype. Consequently, some aspects of modern patterns of human genetic diversity should have been determined by diseases dating from prehistoric times [1]. The clearest example are provided by malaria, which even now affects 500 million people each year and kills some two million. The selective pressure that malaria has imposed to human populations has been reflected in dozens of molecular variants described as protective against the infection and disease [2]–[4]. Of these, the most well studied and widely accepted are probably the sickle cell allele (hemoglobin HbS allele), α and β thalassemias and glucose-6-phosphate (G6PD) deficiency (alleles A and A-), all showing an extensive overlap of geographical distribution and exceptionally high frequencies in malaria endemic regions.

Pyruvate kinase (PK) deficiency, caused by mutations in the pyruvate kinase, liver and RBC (PKLR) gene (chromosome 1q21) is one of the most recently described erythrocyte abnormalities associated to malaria. Evidences of its protective effect were obtained both in murine models [5] and in *Plasmodium falciparum in vitro* cultures using human PK-deficient blood [6], [7]. Also, population studies showed that a selective pressure is shaping the PKLR genomic region in individuals from malaria endemic countries (Cape Verde, Angola and Mozambique), being malaria infection the most likely driving force [8], [9].

PK catalyzes the conversion of phosphoenolpyruvate (PEP) into pyruvate with the synthesis of ATP in the last step of glycolysis. PEP and pyruvate are involved in a great deal of energetic and biosynthetic pathways and the regulation of PK activity has proven to be of great importance for the entire cellular metabolism [10]. PK deficiency, worldwide distributed, is the most common enzyme abnormality in the erythrocyte glycolytic pathway causing hereditary chronic nonspherocytic hemolytic anemia. It is transmitted as an autossomal recessive trait and clinical symptoms usually occur in homozygotes and in compound heterozygotes for two mutant alleles. The clinical phenotype is heterogeneous, ranging from a mild chronic hemolytic anemia to a severe anemia presenting at birth and requiring exchange transfusion [11].

High frequencies of PK deficiency have not yet been recorded in malaria endemic areas but a systematic analysis has never been performed. Considering the previous knowledge of co-distribution between malaria endemicity and protective polymorphisms, we questioned if a PK variant could be exceptionally prevalent in malaria endemic areas. Therefore, the aims of the present study were: i) to determine PK deficiency occurrence in sub-Saharan African countries, ii) to assess frequency of PK variants underlying this deficiency, iii) to investigate possible associations between PK deficiency and malaria infection.

## Materials and Methods

### Sampling

**Figure 1:**
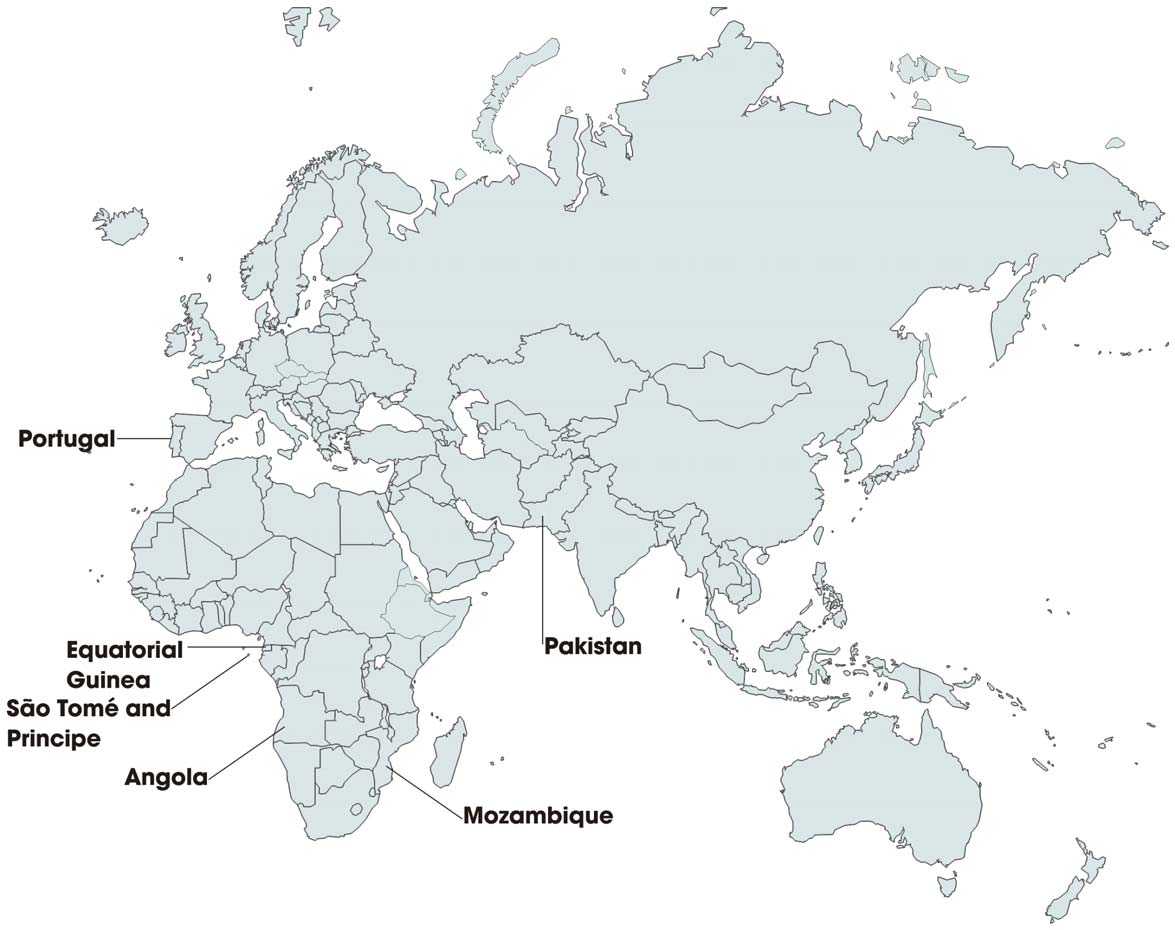
Geographic location of the countries Mozambique, Angola, Sao Tome and Principe, Equatorial Guinea (Africa), Pakistan (Asia) and Portugal (Europe).

This study is based on the molecular analysis of six sets of blood samples collected in four sub-Saharan African areas – Mozambique, Angola, Equatorial Guinea and Sao Tome and Principe (see Figure 1) - and in a malaria non-endemic area – Portugal (Europe).

In this study, 296 unrelated whole blood samples from individuals who attended to the Central Hospital of Maputo (Mozambique) between September and December 2008 were analyzed: 144 from children (6 months to 14 years-old) who presented to the Emergency Services of the Pediatric Department with some kind of complaint, and 152 from healthy blood donor adults (16 to 65 years-old) who presented to the Blood Bank. In order to increase the sample size of the set with a malaria outcome characterization, an additional group of 151 DNA samples extracted from blood samples collected from 3 months to 15 years-old children in Mozambique [9] was also genotyped.

In the Pediatric Department, blood was collected by venous puncture after the clinician examination but before the administration of any anti-malarial drug and/or blood transfusion. The registration of symptoms, axillary temperature and hemoglobin level was done for all individuals. Children who had received a blood transfusion in the last six months were excluded from the study. Anemic and *Plasmodium* infection status were considered at collection time. In the Blood Bank, the blood samples were randomly collected from blood donors. In the admission, a solubility test for rapid detection of hemoglobin S (adapted from Loh [12]) was performed in order to exclude allele S carriers. After blood collection in a tube, a blood spot in a filter paper was prepared from each sample for later subsequent DNA extraction by a standard phenol-chloroform method.

In addition to these samples from Mozambique, a set of 343 DNA samples from malaria-infected and non-infected unrelated individuals, which were already available from other studies, were also analyzed: 164 from Angola [9], 38 from Equatorial Guinea [13] and 67 from Sao Tome and Principe [14]. Finally, 74 samples from non-infected Portuguese individuals from all age groups were used as control samples [8]. Overall, 790 samples were analyzed.

### Ethics statement

Regarding the survey in Mozambique, the human isolates collection was approved by local Ethical Committee (Comité Nacional de Bioética para a Saúde, Health Ministry of Mozambique, IRB 00002657, ref. 226/CNBS/08) and IHMT (Conselho de Ética do Instituto de Higiene e Medicina Tropical, CEIHMT, 14-2011-PN). A detailed work plan, questionnaires and informed consent forms were submitted to the Ethical Committees of the participant institutions in the study, which approved the survey. Each individual and parent/tutor of the children was informed of the nature and aims of the study and was told that participation was voluntary; written informed consent was obtained from each person (or parent/tutor). Blood sample collection followed strict requirements set by the Ethical Committees: blood samples from children who attended to the Pediatric Department were the remaining volume of the samples previously collected for the medical diagnosis; in the Blood Bank, during the blood donation, a small volume was put aside in a tube. In this way, no extra blood collection was needed and the patient, blood donor and the routine health services were not significantly disturbed. All ethical aspects related with the other sets of samples collected in previous studies, are described in the respective reports [8], [9], [13], [14].

### *Plasmodium* infection and malaria outcome groups

In the Central Hospital of Maputo, the rapid test OptiMAL-IT (DiaMed, Switzerland) was used for malaria diagnosis in all the patients with suspicion of malaria infection, and a blood smear was prepared for microscopic visualization to confirm diagnosis; later, all samples were amplified by Polymerase Chain Reaction (PCR), using *Plasmodium* species specific primers [15].

Malaria outcome was defined as follows: (i) Severe Malaria (SM): positive PCR for any species of *Plasmodium*, fever (i.e. axillary temperature ≥37,5°C), hemoglobin level of Hb≤5 g/dL and/or any of these symptoms: coma, prostration or convulsions; (ii) Uncomplicated Malaria (UM): positive PCR for any *Plasmodium* species, fever and hemoglobin level of Hb>5 g/dL; and (iii) Asymptomatic Infection (AI): positive PCR for any *Plasmodium* species in the absence of fever (i.e. axillary temperature <37,5°C) or other symptoms of clinical illness; (iv) No infection (NI): negative PCR and absence of fever or other symptoms of clinical illness.

Based on malaria infection and symptoms data, the 144 samples from the Pediatric Department of Central Hospital of Maputo collected in 2008 were organized in the following malaria outcome groups: SM (18 samples); UM (27 samples) and NI (99 samples). The 152 samples from the Blood Bank were organized in the following groups: AI (4 samples) and NI (148 samples). Outcome groups were also defined using the same criteria for the set of isolates from Angola (43 SM, 43 UM, 37 AI and 41 NI) and for the set of isolates previously collected in Mozambique (52 SM, 97 UM and 2 NI), both described in Machado *et al.* [9]. In total, we had 611 samples with malaria infection and outcome characterization - 459 samples from children (113 SM, 167 UM, 37 AI and 142 NI) and 152 samples from adults (4 AI and 148 NI).

### Determination of PK activity

PK activity was measured in lyzed erythrocytes from all the 296 fresh blood samples (after plasma and buffy coat strict removal) collected in Mozambique in 2008, with an enzymatic assay adapted from Beutler [16], according to the instructions of the kit “Determination of pyruvate kinase (EC 2.7.1.40) in erythrocytes hemolysate or serum/heparinized plasma” (Instruchemie, The Netherlands). The enzymatic reactions were running at room temperature. A PK-deficient and a normal control were used in each assay to validate the activity values and to classify the samples within the following phenotypes: normal, intermediate or deficient activity.

### Identification of a PK variant underlying PK-reduced activity

Samples with a PK activity value less than or equal to 75% of the normal control sample activity were analyzed by the Single Strand Conformational Polymorphism (SSCP) method (described in Manco *et al.* [17]) in order to find a mutation associated with this phenotype. The promoter region and eleven exons of the PKLR gene were amplified with specific primers (see Table S1, supporting information) and run in an acrylamide-bisacrylamide gel (10%), together with a wild-type amplicon, to detect differences in migration patterns caused by an alteration in DNA chain composition (exon 2 was not analyzed since it is specific for the hepatic isoenzyme). The amplification conditions were: initial denaturation at 94°C for 5 minutes, followed by 35 cycles of 94°C for 45 seconds, a specific annealing temperature for 45 seconds (see Table S1), and 72°C for 1 minute, with a final extension at 72°C for 5 minutes. The samples with a different migration pattern were further analyzed by automatic DNA sequencing (Macrogen Inc., Korea). The exon 7, in which a mutation was identified, was then amplified in all samples from all groups by PCR with the specific primers and conditions indicated in Table S1 and the amplicons were sequenced (Macrogen Inc., Korea).

### Statistical analysis

The association between alleles and malaria outcome groups was assessed by Pearson's chi-square tests and Fisher's exact test, this latter considered when there were a few cases in each comparison group (less than five), using the Simple Interactive Statistical Analysis software (SISA) [18]. Odds ratios (OR) and 95% confidence intervals (CI) were also estimated using SISA. Arlequin 3.1 software [19] was used to determine allele frequencies, population pairwise *F_ST_* (to test for differentiation between populations), expected and observed values of heterozygosity and to perform Hardy–Weinberg equilibrium tests. Prediction of the possible impact of the amino acid substitution on the structure and function of the human PK protein was performed with the Polyphen software [20]. Finally, PyMol software [21] was used for the 3D structure simulation of the wild type and mutant variants.

## Results

### PK deficiency screening in Maputo, Mozambique

Ninety-eight from the 144 samples collected in the Pediatric Department (68%) in Mozambique in 2008 were from children with a hemoglobin concentration <9 g/dL (considered anemic) and 41 samples (28.5%) were infected with *P. falciparum*. Nineteen of the infected individuals were also anemic. Four (2.6%) of the 152 samples from the adult blood donors in Blood Bank showed an asymptomatic infection with *P. falciparum* (see Table 1)

**Table 1:** PK activity, anemia and *Plasmodium* infection status in the sample set from Maputo, Mozambique (2008).

	Pediatrics	Blood Bank	Total
**Age Group**	Children (6 months–14 years old); with some complaint	Adults (16–65 years old); healthy blood donors	6 months–65 years old
**Nr of samples**	144	152	296
**Low PK activity (39–75% of control)**	4 (2.8%)	8 (5.3%)	12 (4.1%)
**Anemia (Hb<9 g/dL)**	98 (68.1%)	n.d.	n.d.
***Plasmodium*** ** infection**	41 (28.5%)	4 (2.6%)	45 (15.2%)
**Anemia+Infection**	19 (13.2%)	n.d.	n.d.

n.d.: not determined.

From the 296 samples set, 12 (4.1%) presented PK activity values between 39% and 75% of the normal control activity (established in an average of 3.2 U/g Hb) (see Table 2): 8 from the Blood Bank (5.3%) and 4 from the Pediatrics (2.8%). They were all classified as intermediate activity phenotype. From the 98 samples with a hemoglobin level <9 g/dl (Pediatric Department), only 3 (3.1%) had a PK reduced activity.

**Table 2:** Samples with a reduced PK activity (between 39 and 75% of the normal control) and respective infection status and malaria outcome and 829 locus genotype.

					PK Activity U/g Hb				
#	Sample	Assay	Activity	Average	Control N	Average/Control N	Control DEF	Inf/Malaria outcome	829G/A
1	**BS_128**	1	1.69	**1.69**	3.48	**0.49**	0.85	NI	GG
2	**BS_176**	1	1.88						
	**BS_176**	2	1.93	**1.91**	3.48	**0.55**	0.85	NI	GG
3	**BS_197**	1	1.56						
	**BS_197**	2	1.34	**1.45**	3.48	**0.42**	0.85	NI	**GA**
4	**BS_199**	1	1.73						
	**BS_199**	2	0.99	**1.36**	3.48	**0.39**	0.85	NI	**GA**
5	**BS_212**	1	1.85						
	**BS_212**	2	1.43	**1.64**	3.48	**0.47**	0.85	NI	**GA**
6	**BS_220**	1	1.35						
	**BS_220**	2	1.52	**1.44**	3.48	**0.41**	0.85	NI	GG
7	**BS_230**	1	1.46						
	**BS_230**	2	1.59	**1.53**	3.48	**0.44**	0.85	NI	**AA**
8	**BS_327**	1	1.74						
	**BS_327**	2	1.96	**1.85**	3.48	**0.53**	0.85	NI	GG
9	**N_1159**	1	1.93						
	**N_1159**	2	2.27	**2.10**	2.91	**0.72**	0.73	NI	GG
10	**N_1391**	1	2.19	**2.19**	2.91	**0.75**	0.73	NI	GG
11	**N_1464**	1	1.69	**1.69**	2.91	**0.58**	0.73	NI	GG
12	**O_2341**	1	1.35	**1.35**	2.91	**0.46**	0.73	SM	**GA**

BS: samples collected in the Blood Bank; O and N: samples collected in the Department of Pediatrics; Inf/Malaria outcome: infection status and malaria outcome; 829G/A: 829 genotype; NI: non-infected; SM: severe malaria.

### Identification of a PK variant underlying PK-reduced activity

**Figure 2:**
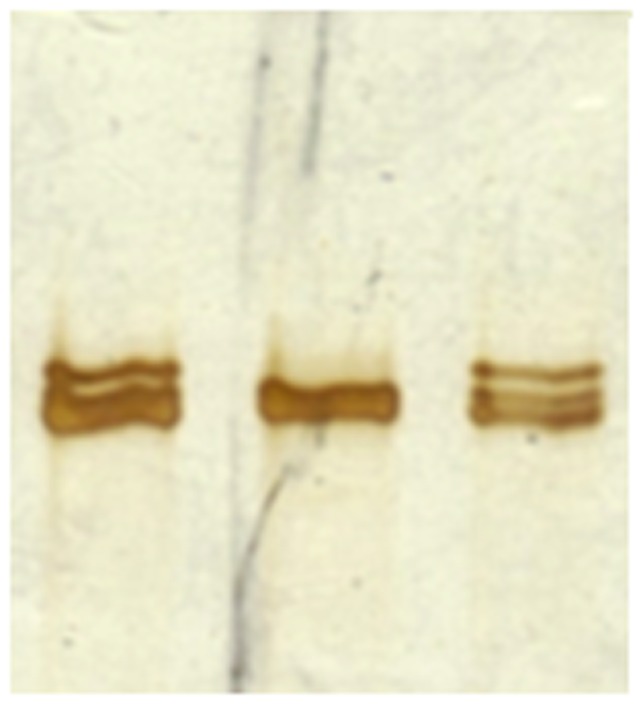
SSCP results showing a migration pattern alteration in the exon 7 amplicons caused by the G829A substitution (10% acrylamide-bisacrylamide gel) - samples at the extremes (wild type isolate in the middle).

A migration pattern alteration was observed in the amplicon of exon 7 of 5 out of 12 samples with low activity (41.7%) by SSCP (see Figure 2): 4 from blood donors and 1 from Pediatrics. Sequencing of these 5 amplicons revealed a G>A substitution in all of them, being in homozygosis (A/A) in one sample. This is a non-synonymous mutation located in the nucleotide 829 of the PK mRNA sequence originating an alteration of the amino acid 277 of the PK protein: a glutamic acid (Glu, coded by GAG) is replaced by a lysine (Lys, coded by AAG). When this mutation was searched in all the other 284 samples with normal activity, it was detected in heterozygosis in 16 samples: 7 from children and 9 from blood donors. Overall, 21 samples (7.1%) had the 829A allele that displayed a frequency of 3.7%.

No association was found between the 829A allele and anemia (2.7–9 g/dL Hb). Conversely, a strong association was found between the allele 829A and PK deficient activity: χ^2^ = 14.38 (P<0.00), OR = 5.58 (95% CI: 2.07–15.03). Of the 6 samples with the lowest PK activity values (between 39% and 47% of the normal activity), 5 had the mutation. All the 6 other samples with an activity between 47% and 75% of the normal activity were wild type.

**Figure 3:**
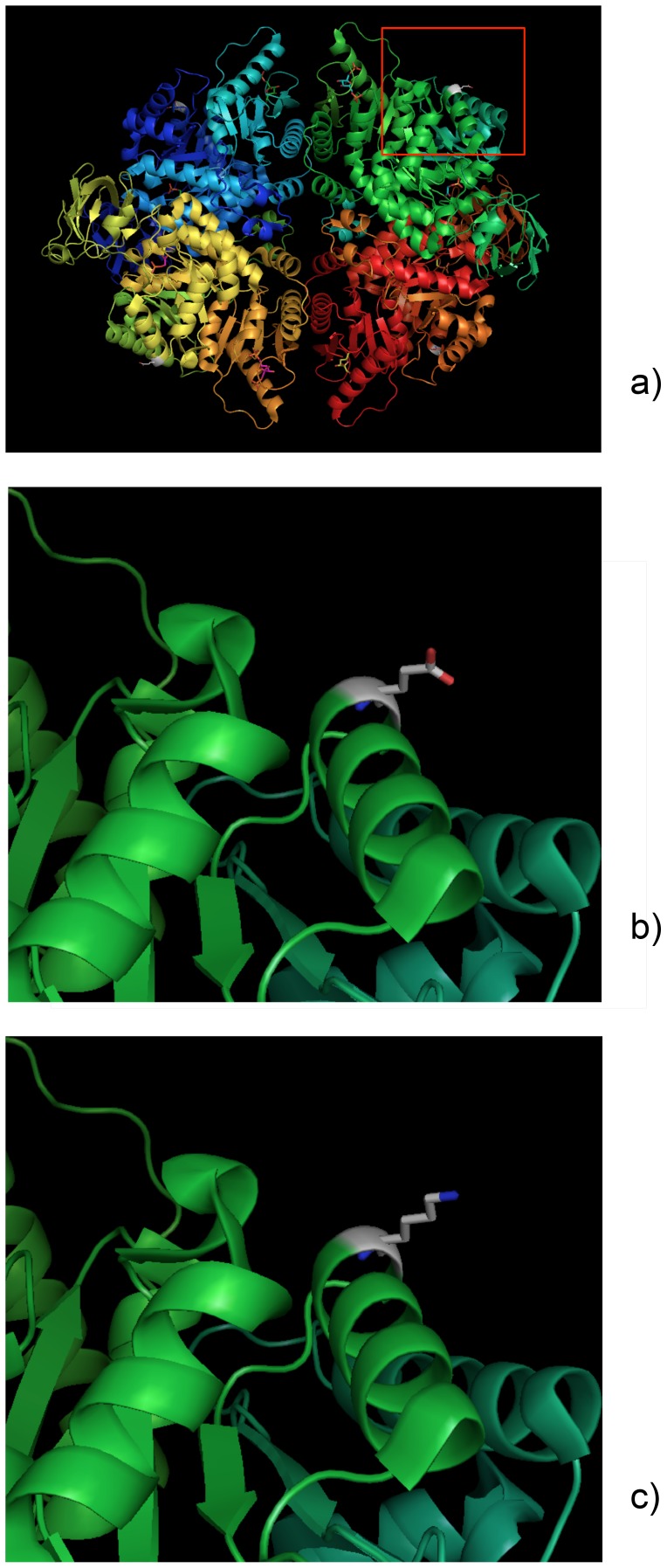
Location of the amino acid 277 in the PK protein and simulation of the 3D wild type 277Glu and mutant 277Lys PK variants structure with the software PyMol. a) Peripheral position of the amino acid 277 (domain A); b) Wild type variant 277Glu; c) Mutant variant 277Lys.

As visualized in the 3D PK structure simulation (see Figure 3), this 277 residue is exposed, showing a peripheral position. The prediction of the substitution Glu277Lys effect on the structure and function of the human protein PK was “Possibly Damaging” (score of 0.90) supporting the previous OR result and suggesting that this mutation is likely to be non-functional.

### Searching the mutation G829A in other African malaria endemic areas

The mutation G829A was found in the other three African countries, always in heterozygosis: in 11 samples from Angola (6.7%), 1 sample from Equatorial Guinea (2.6%) and 2 samples from Sao Tome and Principe (3.0%). Allele 829A frequencies were 3.4%, 1.3% and 1.5%, respectively. In the Mozambican group from 2005, the frequency of individuals heterozygous for 829A was 5.3%, giving an allele frequency of 2.6%. The mutation was not found in the control group from Portugal. Considering all the Mozambican 447 samples, a frequency of carrier individuals of 5.8% and 829A allele frequency of 3.0% were estimated.

The observed genotype frequencies (829GG, 829AG and 829AA) were according to Hardy-Weinberg expectations for all populations (P = 0.40 in Mozambique; P = 1.00 in Angola, Equatorial Guinea and Sao Tome and Principe). Estimates of *F_ST_* were non-significant between all pairs of African populations (*F_ST_*≤0.00 for all) (P = 1.00 for Mozambique vs. Angola; P = 0.50 for Mozambique vs. Equatorial Guinea; P = 0.30 for Mozambique vs. Sao Tome and Principe; P = 0.51 for Angola vs. Equatorial Guinea; P = 0.35 for Angola vs. Sao Tome and Principe; and P = 1.00 for Equatorial Guinea vs. Sao Tome and Principe).

### Association among PK-reduced activity, the mutation G829A and malaria infection/outcome

Six-hundred and eleven DNA samples belonging to individuals characterized for their infection and malaria disease outcome status were analyzed: 459 samples from children (113 SM, 167 UM, 37 AI and 142 NI) from Angola and Mozambique and 152 samples from adults (4 AI and 148 NI), from Mozambique. No significant differentiation between samples from Angola and Mozambique were observed, so all samples together were considered for this analysis.

Allele 829A frequencies were as follows (see Table 3): in children, 3.1% in SM, 3.3% in UM, 2.7% in AI and 2.5% in NI; in adults 4.4% in NI. In terms of malaria infection in children, allele A frequencies were 3.2% in infected and 2.5% in non-infected. In adults, this analysis in terms of infection was not considered due to the low number of infected individuals. Although the mutation frequency was higher in uncomplicated (UM) than in severe malaria (SM) group, no significant association was observed between 829A allele and disease outcome (χ^2^ = 0.02, P = 1.00; OR = 1.07, 95% CI: 0.41–2.80). No significant association was found either between 829A allele and infection (χ^2^ = 0.33, P = 0.57; OR = 1.29, 95% CI: 0.54–3.08) or between PK deficient activity (low enzyme activity) and infection (P = 0.30), though 11 from the 12 samples with PK reduced activity were non-infected.

**Table 3:** Allele 829A frequencies in infection and malaria outcome groups.

	CHILDREN^1^			ADULTS^2^		
Infection/Clinical group	Samples	829A carriers	829A frequency	Samples	829A carriers	829A frequency
**SM**	113	7 (6.2%)	3.1%	0	0 (0%)	0 (0%)
**UM**	167	11 (6.6%)	3.3%	0	0 (0%)	0 (0%)
**AI**	37	2 (5.4%)	2.7%	4	0 (0%)	0 (0%)
**NI**	142	7 (4.9%)	2.5%	148	13^3^ (8.8%)	4.7%
**INF (SM+UM+AI)**	317	20 (6.3%)	3.2%	4	0 (0%)	0 (0%)
**TOTAL**	459	27 (5.9%)	2.9%	152	13 (8.6%)	4.6%

1 Samples from children well characterized for infection and malaria outcome status from Maputo, Mozambique (collected within this study and in a previous one [9]) and from Angola (collected previously [9]) who attended to the Pediatrics Department.

2 Samples from adult blood donors from Maputo, Mozambique (collected within this study).

3 Including one 829AA homozygote (the only one identified in the study).

SM: severe malaria; UM: uncomplicated malaria; AI: asymptomatic infection; NI: non-infected; INF: infected.

## Discussion

This is the first study aimed at determining PK deficiency occurrence as well as at studying a potential widespread PKLR mutation in the African continent.

In the first instance, PK deficiency was studied in samples from Maputo, Mozambique, measuring PK activity in anemic individuals, as this is described as a symptom of the disease. However, anemia was neither associated to PK reduced activity nor 829A allele. The overall prevalence rate of PK reduced activity was 4.1% in the study population (5.3% from blood donors and 2.8% from children). Although children samples were, most of them, clinical cases with a considerable anemic status, a higher PK deficiency prevalence was not found in these samples and no association was detected between PK low activity and anemia. In this regard, a study carried in 2002 revealed that 74% of the children under five and 50% of the women in reproductive age from Mozambique was anemic [22], showing that anemia is not a proper indicator of erythrocyte deficiencies in developing countries.

The missense mutation G829A (Glu277Lys) was identified in 41.7% of Mozambican PK deficient isolates with a strong association with reduced activity phenotype. This mutation was then searched in additional Mozambican samples and other sub-Saharan regions and the 829A allele was detected in all of them at allele frequencies between 1.3% (in Equatorial Guinea) and 3.4% (in Angola). The allele 829A was not present in the Portuguese samples. Although two African groups could be established according to these frequencies (Angola and Mozambique with higher frequencies vs. Equatorial Guinea and Sao Tome and Principe with lower frequencies), *F_ST_* values were not significantly different between them. These differences may be explained by sample size bias (447 samples from Mozambique and 164 from Angola were processed against 38 from Equatorial Guinea and 64 from Sao Tome and Principe) or design bias (isolates from Mozambique and Angola were obtained in hospital-based studies, whereas the others were collected in households by active search). In addition, genetic substructure among geographic regions cannot be excluded as a hypothesis for this disparity. Differences in malaria selective pressure are not a probable cause, since it has probably been similar in all these regions in the past.

Prevalence of PK deficiency seems to vary greatly among ethnic groups and geographic regions, as well as the mutations in the PKLR gene. Some authors have estimated a prevalence of 1∶20 000 in the general white population [23]. In Europe, an incidence of 3.3 per million has been reported in the north of England [24], and a prevalence of 0.24% and 1.1% have been described in Spain [25] and Turkey [26], respectively. In Asia, the frequency of PK deficiency among the Hong Kong Chinese population was <0.1% [27] whilst among the south Iranian population was 1.9% [28]. In Saudi Arabia, a prevalence of 3.12% was registered in newborns [29]. These studies were all based in PK activity measurements. The estimated mutant allele frequencies of common variants generally vary between 0.2 and 0.8% [23] with the highest heterozygous prevalence described so far in Saudi Arabia (6%) [28], [30] and Hong Kong (3.4%) [31]. However, these last allele frequencies were not calculated from mutation genotyping but only estimated from the Beutler's screening qualitative procedure and enzyme assay [16], which result in less reliable estimates of heterozygosity. Moreover, consanguinity is extremely high in Saudi Arabia, exceeding 80% in some regions [29], which tends to bias the results.

The PK deficiency recorded in Mozambique (4.1%) and 829GA heterozygous prevalence (2.6–6.7%) determined from unrelated individuals from sub-Saharan populations is, to our knowledge, the highest estimated worldwide so far. We initially hypothesized that this would be the result of a strong malaria pressure, but a significant association between both PK low activity and 829A and malaria infection and outcome was not found. However, only 12 samples were available for testing a possible effect of low enzyme activity on severity of malaria and 20 samples for testing a possible effect of 829A allele meaning that larger numbers are required to formally conclude. Moreover, since this was a cross-sectional study, infection and malaria outcome groups were established according to a malaria phenotype in a specific time point (the collection day), that may not accurately reflect the true individual phenotype. Nevertheless, there was higher mutation prevalence in the uncomplicated malaria group supporting that further analysis is essential to complete the present study.

The Glu277Lys mutation here identified has been previously reported in the PKLR mutation database [32] and has recently been described [30] in only two individuals: one from the Mandenka ethnic group (one of the largest ethnic groups in West Africa) and other from the Brahui ethnic group from Pakistan, showing that is also present in Middle East. Since the haplotypes that include this mutation in these two individuals are different, it was suggested that it has arisen separately. In Pakistan, as in sub-Saharan countries, malaria continues to be a major public health problem. Both *P. falciparum* and *Plasmodium vivax* are widely distributed and the estimated number of annual malaria episodes in this country is 1.5 million [33].

The simulation of this Glu277Lys substitution on the human PK protein suggested that this mutation is likely to be non-functional. This residue is extremely well conserved and the result complies with the prediction from SIFT from a previous work [30]. Probably, the charge change (Glu is negatively whereas Lys is positively charged) at an exposed site alters the enzyme action. Considering this result together with the knowledge about PK deficiency that clinical symptoms usually occur in homozygotes for a mutant PKLR allele, it was surprising to find that the 829AA genotype belonged to a healthy blood donor without anemia symptoms, with a PK activity of 0.44 with regard to the normal control. In this case we were expecting an activity similar to the deficient control sample (0.8 U/g Hb). However, the results obtained regarding PK activity must carefully be considered since the range of values obtained in Mozambique was narrow, far below the values expected with the use of the kit and generally obtained in other labs (about 3.7–8.2 U/g Hb at 25°C and about 7.4–16.4 U/g Hb at 37°C), with a thin gap between normal and reduced activity. This can be explained by the lower room temperature in the lab (about 20°C), when compared to those generally maintained in this procedure (25°C or 37°C). Yet, the procedure was efficient since it was possible to identify samples with reduced activity. Actually, there was no direct relation between the genotype and phenotype: although a significant association between 829A and a reduction in the enzyme activity was found out (and the samples with the lowest activity were those ones with the 829A allele), the phenotype of allele A carriers was highly variable with a large number of individuals within normal PK activity range. A previous study emphasizes the difficulty in predicting the consequences of mutations simply from the location and the nature of the target residues [10]: the clinical manifestations of a genetic disease reflect the interactions of a variety of physiological and environmental factors, including genetic background, concomitant functional polymorphisms of other enzymes, posttranslational or epigenetic modifications, ineffective erythropoiesis and differences in splenic function, and do not solely depend on the molecular properties of the altered molecule.

To conclude, a geographical co-distribution between malaria and PK-deficiency seems to occur: the Middle East and sub-Saharan Africa are the regions with the highest PK deficiency prevalence described so far, as determined in the present study. These are regions with a strong malaria pressure, suggesting that malaria may be an agent of contribute to the selection of PK deficiency variants in these regions. Conversely, the prevalence of PK deficiency is extremely low in the general white populations. Moreover, some of the genes that confer resistance to malaria are among the most variable genes in the human genome [4] and this is the case for PKLR gene, which presents more than 180 mutations and 8 polymorphic sites [11].

Additional studies with a larger sampling effort including longitudinal malaria clinical history characterization and a search of the variant 277Lys in other malaria endemic regions will be conducted to clarify the results in this survey.

## Supporting Information

Table S1 **List of primers and annealing temperatures (a.t.) used in the amplification of** ***PKLR*** **promoter (Prom) and coding regions by PCR.**
(DOCX)
