# Supplementary material for: Pyruvate Kinase Deficiency in Sub-Saharan Africa: Identification of a Highly Frequent Missense Mutation (G829A;Glu277Lys) and Association with Malaria
Source: PLoS One. 2012 Oct 17;7(10):e47071. doi: 10.1371/journal.pone.0047071 (PMC3474807; doi:10.1371/journal.pone.0047071)
Supplement: Table S1 — List of primers and annealing temperatures (a.t.) used in the amplification of PKLR promoter (Prom) and coding regions by PCR. (DOCX) [file pone.0047071.s001.docx]

# Supporting information

**Table S1**. List of primers and annealing temperatures (a.t.) used in the amplification of *PKLR* promoter (Prom) and coding regions by PCR.

| Exon | Product (bp) | Forward Primer (5’-3’) | Reverse Primer (5’-3’) | PCR a.t. (ºC) |
| --- | --- | --- | --- | --- |
| Prom/1 | 495 | AGCTAACTTCAGTAAAGTAC* | GATGTGGATCATTTATGC | 54 |
| 3 | 286 | GGTGACATGCAGTCCCTGA | AGATGAAGAAGCACCTCAAG* | 56 |
| 4 | 253 | CGTTCTGAGAATGGTAATGG* | GAGGGTTTCAGGGGAAGGT | 60 |
| 5 | 239 | CCACCTTCCCCTGAAACC | CTGGGCCCAACCCTACAG | 54 |
| 6 | 304 | ACTCCGGGGCTCAGAACT | CTGATGGGGGAGCCAAGG* | 62 |
| 7 | 350 | ACCGCAGCTGGCTCTTTC* | GTGATGGGGAATAGCGACAG | 60 |
| 8 | 252 | CACCTTTCTTCTCCTGCCTG | CAGGTGTCCCTAAAACCCAC | 60 |
| 9-10 | 500 | CAGTGTGAGTCCTACAAC* | CTGACCCAAAGCTCCATC* | 56 |
| 11 | 413 | AGTGACACCTGGAACTGG* | GATATCTCAGTCTTAGTG* | 52 |
| 12 | 259 | CCTTGGCTTCCCAAAGTG* | GCTGGAGAACGTAGACTG* | 60 |

*Described in Lenzner *et al*. 1994.

(Lenzner C, Nürnberg P, Thiele BJ, Reis A, Brabec V, et al. (1994) Mutations in the pyruvate kinase L gene in patients with hereditary hemolytic anemia. Blood 83: 2817-2822.)
